# Enhancing AES image encryption with a three-dimensional hyperchaotic system for increased security and efficiency

**DOI:** 10.1371/journal.pone.0328297

**Published:** 2025-07-18

**Authors:** Mingyi Huo, Yanpei Zheng, Jun Huang

**Affiliations:** 1 College of Information, Mechanical and Electrical Engineering, Shanghai Normal University, Shanghai, China; 2 Information Network Company of Gansu Civil Aviation Airport Group Co., Ltd., Lanzhou, China; 3 College of Information Science and Engineering, Lanzhou University, Lanzhou, China; University of Buner, PAKISTAN

## Abstract

In the digital era, the security of images, as critical carriers of information, is paramount for national security, military strategy, and personal privacy protection. Therefore, developing efficient and cost-effective image encryption technologies to ensure the security of image data during transmission has become an urgent necessity. Although the Advanced Encryption Standard (AES), a widely used symmetric encryption method, performs excellently in data communication and network security, its efficiency and security face significant challenges when directly applied to image encryption due to the inherent complexity of image data. This paper presents a simplified AES image encryption framework based on a three-dimensional hyperchaotic system (TDHCS). The crux of this framework is the incorporation of a novel TDHCS, distinguished by its intricate nonlinear dynamics and robust randomness. The AES encryption process is simplified based on the high-level random chaotic sequences generated by TDHCS. In contrast to the traditional fixed S-box, a dynamic S-box generation scheme is employed, while a random operation scheme is proposed as a substitute for fixed sequences in the round key addition and row shifting steps. Experimental results show that the proposed algorithm reduces encryption time to 2.2369 s for a 256×256 grayscale image, representing an 87.08% improvement over traditional AES (17.3090 s) and a 48.41% improvement over other AES-based chaotic encryption algorithms (4.3360 s). Furthermore, the security analysis demonstrates that the algorithm effectively resists differential attacks, achieving NPCR, UACI, and BACI values with deviations of only 0.0031%, 0.0046%, and 0.0288%, respectively. The proposed algorithm markedly reduces the number of encryption rounds to one while enhancing both efficiency and security. Simulation results confirm its robustness against various cryptographic attacks, demonstrating its potential as a preferred solution for digital image privacy protection.

## 1 Introduction

In the context of rapidly advancing technology, data security has ascended to unprecedented strategic importance. Particularly with the frequent occurrence of large-scale data breaches [1], the protection of sensitive information has become a focal point of concern across various sectors of society. As one of the key technologies addressing this challenge, the Advanced Encryption Standard (AES) has firmly established itself as a cornerstone in modern cryptographic systems [2,3], owing to its exceptional encryption strength, broad compatibility, and efficient implementation across diverse platforms [4–6]. However, as digital images are increasingly employed in fields such as aerial reconnaissance, military communications, and the protection of commercial secrets, the secure transmission and storage of image data has emerged as a new area of research [7–9]. Compared to structured text data, digital images, due to their high redundancy, large volume, and complex spatial correlations, impose stricter demands on the efficiency and security of encryption algorithms [10]. While AES demonstrates outstanding performance in protecting textual data, its direct application to image encryption may encounter issues such as low encryption efficiency, or insufficient adaptability to encryption modes [11–13]. Therefore, the customization and optimization of the AES algorithm, tailored to the unique characteristics of image data, has become a critical problem that urgently needs to be addressed in the field of cryptography [14].

Chaos theory, with its unique behavior of determinism in states of uncertainty and high sensitivity to initial conditions, offers a powerful tool for image encryption [15–17]. The characteristics of these nonlinear systems align closely with the demands of image encryption, particularly when handling complex and dynamic image data [18,19]. In earlier research, Muhaya *et al*. [20] first applied an Arnold cat map to shuffle the image pixels, followed by using a chaotic Henon map to generate the AES encryption key. The improved AES algorithm achieved a sufficiently large key space and enhanced key sensitivity. In literature [21], the fusion of chaos theory and image encryption technology is explored, proposing an innovative image encryption scheme. This scheme utilizes the Zhongtang chaotic system to generate the S-box used in the byte substitution process, and completes the encryption of the entire image data through three rounds of encryption iterations. In 2021, Shariatzadeh *et al*. [22] leveraged the complexity and unpredictability of the Logistic chaotic system to generate algorithmic keys for AES. These keys were used to create four highly random images, playing a critical role in enhancing the round key addition process of AES. They proposed a dynamic AES encryption algorithm with only three encryption rounds, significantly improving the security of AES in the field of image encryption. Liu *et al*. [23] proposed a dynamic AES cryptosystem based on a memristor neural network, which introduces memristors into a transient chaotic neural network for initial key generation. The system dynamically alters the encryption key for each instance based on chaotic sequences, achieving a “one-time pad” encryption approach. Compared to traditional AES, the proposed algorithm demonstrates enhanced security, a larger key space, and greater robustness. Although numerous studies have focused on improving the encryption efficiency and security of AES, several challenges persist in practical applications. First, existing approaches primarily rely on chaotic sequences generated by chaotic systems to enhance the randomness of the encryption process, aiming to reduce the number of encryption rounds and thereby increase efficiency. However, the chaotic systems used in these approaches often exhibit insufficient chaotic behavior and suboptimal randomness, necessitating multiple encryption rounds to ensure security [24]. Furthermore, several steps in the AES encryption algorithm involve fixed parameters, and current methods have only dynamically modified some of these steps using chaotic systems, limiting the potential for fully leveraging the randomness of chaotic systems to significantly enhance security.

To address the aforementioned challenges, this paper proposes an improved AES encryption algorithm based on a three-dimensional hyperchaotic system. Firstly, a novel three-dimensional hyperchaotic system is introduced, and subsequently, several steps in the AES encryption algorithm that involve fixed parameters are modified dynamically using this system. This algorithm not only extends the application of chaotic systems in AES, but also significantly enhances the encryption efficiency and security of the AES algorithm. The main contributions of this paper are as follows:

1. We propose a new three-dimensional hyperchaotic system (TDHCS), which, in comparison to traditional one-dimensional or two-dimensional chaotic systems, displays more intricate dynamic characteristics and more pronounced nonlinear behaviour. In particular, the TDHCS system exhibits positive Lyapunov exponents in all three dimensions, thereby greatly enhancing the unpredictability of its phase space trajectories. This provides a more robust chaotic foundation for the encryption algorithm.
2. We explore the deeper application of chaotic systems in the AES algorithm by applying the randomness of chaotic sequences to the generation of the S-box, round key addition, and row shifting stages. By replacing the fixed parameter encryption steps of the original AES with dynamic operations, encryption can be completed in just one round, improving both the efficiency and security of the AES algorithm, making it more suitable for protecting image data.

The structure of this paper is as follows: Sect 2 introduces the TDHCS and analyzes its chaotic characteristics using bifurcation diagrams, phase diagrams, and Lyapunov exponents, comparing it with existing chaotic models. Sect 3 provides a detailed description of the image encryption algorithm. Sect 4 analyzes the simulation results of the algorithm. Finally, Sect 5 summarizes the findings and conclusions of the study.

## 2 A three-dimensional hyperchaotic system

In this paper, we introduced a three-dimensional hyperchaotic system (TDHCS) utilizing three ternary linear equations. By employing modular operations, this system functions as a folding mechanism. The state of this three-dimensional discrete chaotic system is driven and operated by the interaction of different variables, resulting in complex dynamic behavior at the system level. Compared to low-dimensional chaotic systems, three-dimensional hyperchaotic systems exhibit more intricate nonlinear dynamic behaviors, characterized by strong uncertainty and randomness. The three-dimensional hyperchaotic system model used in this paper exemplifies these advanced dynamics. The mathematical expression of the model is shown in Eq 1.

$$(x_{n+1},y_{n+1},z_{n+1})=F(x_{n},y_{n},z_{n})=\left\{ \begin{array}{c} mod(\alpha *z_{n},1)\\ mod(\beta *x_{n},1)\\ mod(\gamma *y_{n}-x_{n},1) \end{array}\right.$$ (1)

where *x*, *y*, *z* are state variables, α, β, and γ are control parameters, and cannot be zero at the same time. In our experiments, we set these parameters to α=5, β=24, and γ=23 to ensure the best chaotic performance.

To comprehensively evaluate the performance of hyperchaotic systems, this study conducts a series of experiments and employs advanced analytical techniques, including the Lyapunov exponent, bifurcation diagram, phase diagram, and entropy diagram, to assess system stability and complexity. The results are then compared with those of recent two-dimensional chaotic systems, such as the cross two-dimensional hyperchaotic map (C2HM) [25] and the two-dimensional Logistic-Sine coupling map (2D-LSCM) [26].

The chaotic system of C2HM is shown in Eq 2.

$$\left\{ \begin{array}{c} x_{n+1}=sin[\frac{\alpha }{sin\left(y_{n}\right)}]\\ y_{n+1}=\beta sin\left[\pi \left(x_{n}+y_{n}\right)\right] \end{array}\right.$$ (2)

where its control parameters α≠0, β∈(0,1]. The initial value $y_{0}\neq 0$, the system acquires the best chaotic performance at α∈(0,2] and β∈(0,1]. In our experiments, we set α=2 and β=1, following the optimal values reported in the original literature.

The chaotic system of 2D-LSCM is shown in Eq 3.

$$\left\{ \begin{array}{c} x_{n+1}=sin(\pi (4\theta x_{i}(1-x_{i})+(1-\theta )sin(\pi y_{i})))\\ y_{n+1}=sin(\pi (4\theta y_{i}(1-y_{i})+(1-\theta )sin(\pi x_{i+1}))) \end{array}\right.$$ (3)

where θ is the control parameter and θ∈(0,1]. For our experiments, we used θ=0.99, as suggested in the original study to ensure optimal chaotic behavior.

### 2.1 Lyapunov exponent analysis

The Lyapunov exponent (LE) reflects the sensitivity of the system to initial conditions, helping to determine whether the system exhibits chaotic behavior and to understand the degree of chaos. By calculating the Lyapunov exponent for different trajectories in the system, we can conduct a comprehensive quantitative analysis of the system’s dynamic characteristics.

**Fig 1:**
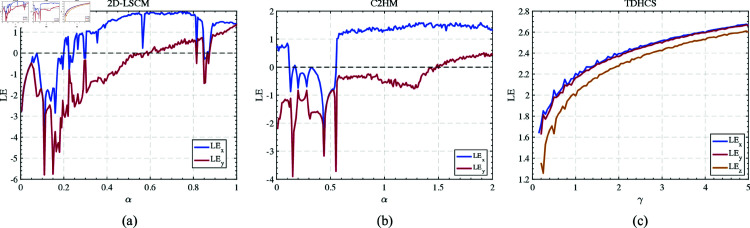
Lyapunov exponent of different chaotic maps. (a) 2D-LSCM. (b) C2HM. (c) TDHCS.

Fig 1 compares the LE diagrams of TDHCS with two other systems, where the parameter γ is used as a control variable. Within the defined parameter range, the LE values of the TDHCS show steady growth and stability, outperforming the other two systems in dynamic behavior and robustness. Within the specified parameter range, the LE in all three directions exhibit a positive growth trend as γ increases, indicating hyperchaotic behavior. This suggests that our system demonstrates strong unpredictability and complexity, which enhances its resilience to phase space reconstruction attacks. Furthermore, the system requires fewer multiplicative operations, reducing its overall computational complexity.

### 2.2 Bifurcation diagram analysis.

**Fig 2:**
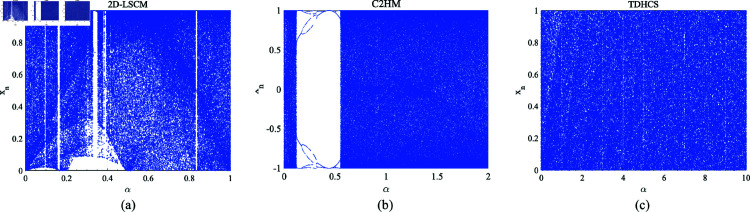
Bifurcation diagram analysis results. (a) 2D-LSCM. (b) C2HM. (c) TDHCS.

Bifurcation plots are indispensable tools for observing how changes in system parameters influence system behavior. By plotting system trajectories in parameter space, researchers can track occurrences of period-doubling phenomena across various parameter values, thereby investigating system bifurcation behaviors and exploring its dynamic properties. Fig 2 illustrates the bifurcation diagram of three chaotic systems as their control parameters increase. The diagram reveals that the three-dimensional chaotic system introduced in this study sustains a chaotic state throughout the entire parameter range, with evenly distributed chaotic states and no noticeable clustering, underscoring the system’s robust chaotic behavior.

At the same time, 2D-LSCM and C2HM exhibit different periodic states and uneven distribution in the parameter range. Therefore, it is obvious that the chaotic range of TDHCS is uniformly distributed and there is no obvious clustering of chaotic states within its parameter range, which emphasizes the robust chaotic behavior of the system.

### 2.3 Phase diagrams analysis

**Fig 3:**
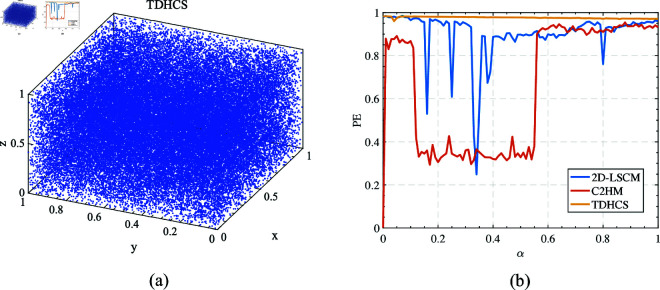
Phase diagram analysis results and alignment entropy analysis results. (a) Phase diagram of TDHCS. (b) Permutation entropy values of the three chaotic systems.

The chaotic behavior of chaotic systems can be intuitively evaluated through bifurcation diagrams and phase diagrams. Fig 3(a) presents the three-dimensional phase diagram of the chaotic system, plotted using state variables (*x*,*y*,*z*) as coordinates. The initial conditions are set to *x*_0_ = 0.1, *y*_0_ = 0.2, *z*_0_ = 0.3, and simulations are conducted over 35,000 time steps. The system’s trajectory covers the entire bounded space extensively without displaying any periodic patterns, indicating a high degree of chaotic behavior.

### 2.4 Permutation entropy analysis

Permutation entropy (PE) [27] is used to quantify the regularity and complexity of dynamic systems. A higher PE value indicates greater irregularity and complexity within the system. Fig 3(b) compares the entropy of our system with two traditional chaotic systems, the C2HM and the 2D-LSCM . As can be seen from the results, our system achieves a PE value closer to the theoretical maximum and exhibits greater stability compared to the other systems. This indicates the superior chaotic performance of the proposed system, which is capable of generating more chaotic, unpredictable, and robust random sequences.

## 3 Enhancing AES image encryption

**Fig 4:**
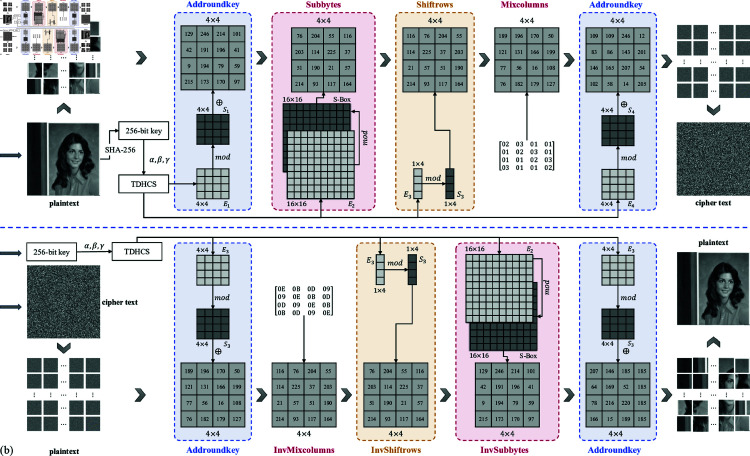
Algorithm flowchart. (a) Flowchart of the encryption algorithm. (b) Flowchart of the decryption algorithm.

Cryptography abides by Kerckhoffs’s principle [28], stipulating that the encryption algorithm’s security hinges solely on the secrecy of the key, while the encryption process and steps remain public. A robust encryption algorithm necessitates a secure key capable of withstanding various cryptographic analyses, alongside transparent encryption steps. Therefore, the selection and generation of keys are pivotal in encryption algorithms. Using a fixed key significantly undermines algorithmic security, rendering the key vulnerable to exploitation. To fortify against decryption and reversal, this study employs the SHA-256 algorithm to derive a HASH value from the plaintext image for encryption. Subsequently, an initial value generation algorithm derives control parameters for a hyper chaotic system from this HASH key. These parameters control inputs into the TDHCS to generate a fixed-length random sequence, pivotal in two aspects of the encryption process. Firstly, it defines operations in AES encryption, such as establishing a row displacement process guided by chaotic sequences. Secondly, it supplements encryption operations by XORing with plaintext content during round key additions. Chaotic sequences inject randomness into data and encryption processes, enabling the algorithm in this study to complete image encryption in a single round while ensuring high security. Refer to Fig 4(a) for details on the encryption process.

This encryption algorithm comprises three main steps:

(1) Key generation: Utilizing a key generation algorithm, the encryption key is derived from the image file slated for encryption. The key exhibits high correlation with the plaintext image content, ensuring that even minor alterations in the plaintext image produce significant changes in the key.

(2) Chaos sequence generation: Following key generation, the chaotic system’s initial parameter generation method determines control parameters and initial values for TDHCS from the encryption key. Through iterations of the hyperchaotic system, a random sequence of specified length is generated. To mitigate transient effects, an initial segment of the chaotic sequence is discarded, and the remainder is employed in the encryption process.

(3) Image encryption: In this step, traditional AES key expansion is substituted with a random sequence generated by the hyperchaotic system. The encryption process mirrors traditional AES methodology, encompassing five operations in a single encryption round: round key addition, byte substitution, row shifting, column mixing, and final round key addition. Leveraging the high sensitivity and randomness of chaotic systems, the simplified AES encryption algorithm achieves completion in just one encryption round.

### 3.1 Secret key structure

SHA-256 is a secure hash algorithm standardized by the US National Security Agency under Secure Hash Algorithm 2 [29,30]. In this study, SHA-256 is employed to compute a 256-bit hash value from the image designated for encryption, serving as the encryption algorithm’s key. The initial parameters of the TDHCS encompass three control parameters and three initial values. To ensure diverse parameter generation, the encryption key undergoes segmentation during calculation, followed by separate processing. This segmented approach not only enhances diversity but also ensures that changes in key positions directly influence the chaotic system’s control parameters, thereby indirectly impacting the encryption outcomes. Consequently, the process of calculating initial parameters integrates segmented key results, facilitated by the generation of intermediate variables.

**Input:** Image to be encrypted.

**Output:** Initial parameters of chaotic system α, β, γ, $x_{1},y_{1},z_{1}$.

**Step 1:** Hash value acquisition and segmentation: Utilize the SHA-256 algorithm to compute the hash value *T* of the image intended for encryption, and obtain a binary number with a length of 256. Divide it evenly into 32 binary numbers, denoted as *T*_1_, *T*_2_ ··· *T*_32_.

**Step 2:** Segmentation calculation: Generate four decimal numbers *K*_1−4_ ithin the range of 0 to 1 based on the values derived from *T*_1−32_. These decimals serve as intermediate variables for computing the initial parameters of the chaotic system. The calculation method is detailed in Eq 4.

$$\left\{ \begin{array}{c} K_{1}=\frac{T_{1}⊕T_{2}⊕\cdot \cdot \cdot ⊕T_{8}}{255}\\ K_{2}=\frac{1}{2}K_{1}+\frac{T_{9}⊕T_{10}⊕\cdot \cdot ⊕T_{16}}{2\times 255}\\ K_{3}=\frac{1}{2}K_{2}+\frac{T_{17}⊕T_{18}⊕\cdot \cdot ⊕T_{24}}{2\times 255}\\ K_{4}=\frac{1}{2}K_{3}+\frac{T_{25}⊕T_{26}⊕\cdot \cdot ⊕T_{32}}{2\times 255} \end{array}\right.$$ (4)

where ⊕ represents XOR operation. According to the formula, *K*_1_ is determined by the first 64 bits of the key, *K*_2_ by the first 128 bits, *K*_3_ by the first 192 bits, and *K*_4_ by the entire key.

**Step 3:** Combination calculation: To ensure strong correlation between the initial parameters of the chaotic system and the key, a combination calculation involving *K*_1−4_ is employed to derive the initial parameters used in the chaotic system. This method ensures that any alteration in the key directly impacts the chaotic system’s initial parameters, thereby modifying the entire encryption process and bolstering the algorithm’s resilience against external attacks. The calculation method for determining the initial parameters of the chaotic system is detailed in the following equation:

$$\alpha =\overset{―}{\alpha }+\frac{mod[(h_{1}+h_{2})*10^{14},255]}{256}\;\beta =\overset{―}{\beta }+\frac{mod[(h_{1}+h_{3})*10^{14},255]}{256}\;\gamma =\overset{―}{\gamma }+\frac{mod[(h_{1}+h_{4})*10^{14},255]}{256}\;x=\overset{―}{x_{0}}+\frac{mod[(h_{2}+h_{3})*10^{14},255]}{256}\;y=\overset{―}{y_{0}}+\frac{mod[(h_{2}+h_{4})*10^{14},255]}{256}\;z=\overset{―}{z_{0}}+\frac{mod[(h_{3}+h_{4})*10^{14},255]}{256}\;$$ (5)

where *mod*(*x*) represents the modulo operation. From the calculation formula, it is evident that any alteration in any of the 256-bit keys will lead to changes in the six control parameters of the chaotic system. Due to the high sensitivity of these initial parameters, any modification will impact the entire encryption process significantly.

### 3.2 Random sequence generation

Input the initial parameters generated by the key into the TDHCS hyperchaotic system for iteration, totaling *G* iterations. To mitigate transient effects, the generated sequence length must be truncated from its starting position and discarded, while ensuring the sequence length meets the encryption requirement. The calculation method for *G* is shown in Eq 6.

$$G=G_{0}+\frac{len(P)}{16}\times 36+256$$ (6)

where *G*_0_ is a constant representing the length of the sequence to be discarded, and *len*(*P*) represents the total number of pixels in plaintext *P*. In this paper, *G*_0_ is set to 2000.

### 3.3 Addroundkey

**Fig 5:**
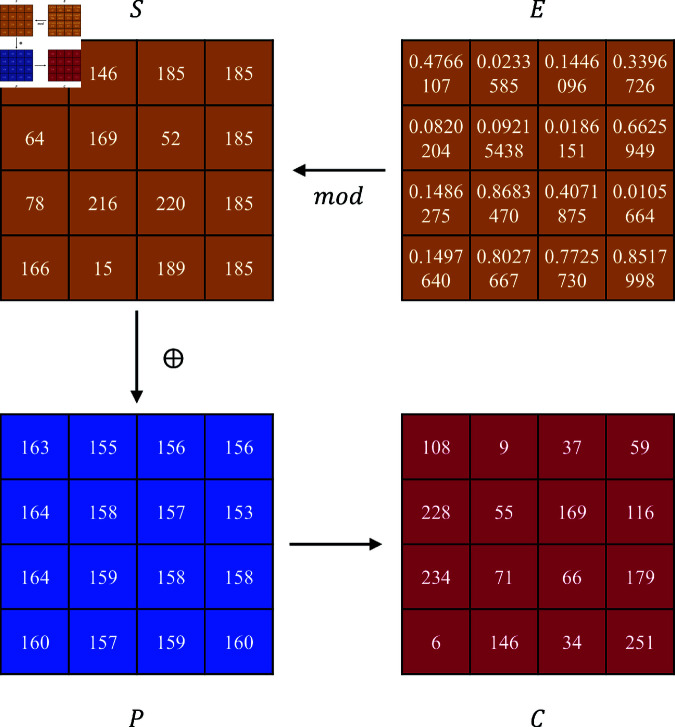
Example of addroundkey process.

In traditional AES encryption algorithms, the data used for XOR operation with plaintext during the round key addition process typically comes from key expansion, which may exhibit poor randomness. In this study, a random decimal sequence generated by a hyperchaotic system is transformed into an integer sequence ranging from [0, 255]. Subsequently, XOR encryption is applied to the image block being encrypted based on its corresponding position. The method for generating random integer sequences is illustrated in Eq 7.

$$S=mod(E\times 10^{16},255)$$ (7)

where *E* denotes a 4*4 random decimal array extracted from a chaotic sequence. The sequence *S* obtained from the chaotic system is encrypted with plaintext *P* at corresponding positions using XOR encryption to produce the result *C* for round key addition. Fig 5 illustrates an example of using chaotic sequences to complete the round key addition process.

### 3.4 Byte substitution

The S-box used in the AES encryption process is a two-dimensional array of 16×16 consisting of 256 positive integers with values ranging from 0 to 255, ensuring no duplicate elements. Serving as a crucial non-linear data structure, S-boxes obscure data during encryption and are integral to AES security. Traditional AES algorithms employ fixed S-box generation methods that remain unchanged regardless of data type, posing security risks. Reference [21] proposes an S-box generation algorithm based on chaotic systems. Initially, a long chaotic sequence is generated using a chaotic system. This sequence is then traversed to convert random decimals into integers within the range [0,255], carefully ensuring no duplicates are added to the S-box during construction.

While this method enhances S-box randomness compared to traditional approaches, its stability and efficiency may be insufficient, potentially wasting significant random sequences. Therefore, this study proposes a novel S-box generation method based on chaotic sequences. This method requires 256 random decimal sequences to construct an S-box with robust non-linear characteristics and no duplicate entries. The detailed steps for generating S-boxes and performing byte substitution are outlined below:

**Input:** A plaintext block *P* of size 4×4 to be encrypted, and a random number sequence *E* of length 256.

**Output:** Encryption result *C* of byte substitution.

**Step 1:** Arrange the random number sequence *E* in ascending order and record the position sequence *E* the sorted numbers in *E* before sorting. In MATLAB, this can be performed using the statements in the following equation:

[E, S] = sort(E) (8)

where *sort*(*x*) performs the sorting operation. Since the sequence numbers in the sorted array are unique and the sorting result is determined by the magnitude of the random numbers in the random array, the elements in the resulting S-box are non-repetitive and random, exhibiting excellent nonlinear characteristics. Adjust the sequence *S* into a two-dimensional array of size 16×16 obtain the S-box. A typical S-box is shown in Table 1.

**Table 1:** Example of S-box.

low four bits																	
high four bits		0	1	2	3	4	5	6	7	8	9	10	11	12	13	14	15
	0	35	70	204	51	252	227	65	62	69	93	213	105	98	22	168	221
	1	135	66	171	160	123	61	142	256	9	121	75	13	80	97	43	18
	2	95	175	241	238	169	5	92	207	185	163	154	38	158	152	24	46
	3	125	7	42	39	179	91	228	29	11	104	55	106	88	203	235	217
	4	78	89	245	120	199	81	6	253	107	190	162	165	37	102	141	208
	5	242	136	76	255	174	28	4	83	230	147	172	153	111	148	198	85
	6	16	243	113	50	57	186	209	248	34	232	233	181	202	116	226	145
	7	14	84	177	74	244	54	180	124	60	222	64	1	250	149	196	130
	8	108	20	156	87	140	157	41	218	32	73	201	215	44	115	225	146
	9	191	219	49	214	126	58	137	52	112	99	94	21	114	127	184	129
	10	138	220	45	197	176	82	237	103	224	110	188	195	170	216	122	134
	11	131	210	12	194	236	109	247	100	151	189	182	173	53	178	139	2
	12	187	33	251	101	48	164	223	200	68	72	155	59	132	30	86	167
	13	192	144	206	47	27	23	40	159	31	205	150	19	249	17	79	231
	14	246	133	254	90	118	240	71	63	3	119	10	143	26	183	234	239
	15	77	166	8	56	211	193	229	25	15	117	128	161	96	67	212	36

**Fig 6:**
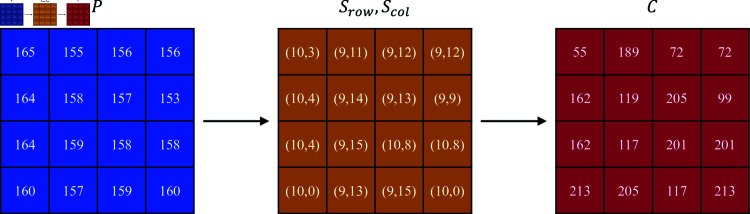
Example of byte substitution Process.

**Step 2:** Generate an S-box based on the chaotic sequence for obfuscation of the encrypted image. For a 256-level grayscale image, each pixel value in the image to be encrypted can be represented as an 8-bit binary number. The upper four bits of the binary value are used as the row number *S*_*row*_ of the replacement target in the S-box, while the lower four bits represent the column number *S*_*col*_ of the replacement target in the S-box. Extract the specified coordinate value from the corresponding position of the S-box and replace it with the pixel value of the image to be encrypted. Fig 6 shows the results of byte substitution of plaintext *P* using the S-box in Table 1.

### 3.5 Shift rows

In the traditional AES algorithm, the rule for row displacement is fixed: the first row remains unchanged, the second row is rotated left by 1 bit, the third row by 2 bits, and the fourth row by 3 bits. This static operation reduces the strength of the encryption, making it more susceptible to attacks. In the algorithm proposed in this chapter, chaotic sequences are used to dynamically generate movement rules for row displacement. The specific generation steps are as follows:

**Input:** A chaotic sequence *E*_*r*_ of length 4, a size of 4×4 image blocks *P* to be encrypted.

**Output:** 4×4 image block C encrypted by row displacement.

**Step 1:** Convert the chaotic sequence *E*_*r*_ into an integer sequence and apply the modulo operation to generate an integer sequence *S*_*r*_ of length 4, where each element has a value range of [0,3]. The calculation method is as follows:

$$S_{r}=mod(E_{r}\times 10^{16},4)$$ (9)

**Fig 7:**
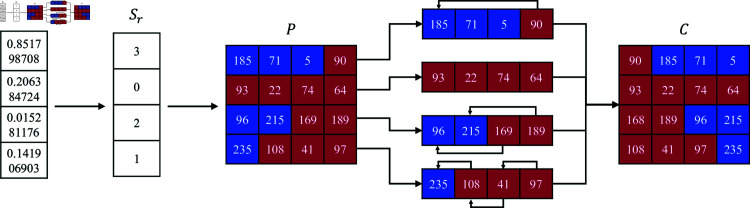
Example of shift rows encryption.

**Step 2:** The integer sequence *S*_*r*_ represents the number of bits to be shifted in each row of the image block *P*, traverse *S*_*r*_ to perform a cyclic shift on *P*. The result of the cyclic shift can be obtained using the method in the equation:

$$C(i)=P(i)[1+S_{r}(i):4]+P(i)[1:S_{r}(i)]$$ (10)

where i∈[1,4] indicates the row index within the image block *P*, where *P*(*i*) denotes the $i_{\text{th}}$ row of this block. The term *P*(*i*)[*x*,*y*] represents the sequence formed by columns *x* through *y* within the $i^{\text{th}}$ row. The symbol + denotes sequence concatenation, combining two sequences into a new, extended sequence. Fig 7 illustrates the outcome of dynamic row displacement encryption based on chaotic sequences, demonstrating the effect on image block arrangement.

### 3.6 Decryption process

While the output sequence of a chaotic system is random, identical initial parameters yield identical outputs. This property of chaos underpins the decryption feasibility of the algorithm presented in this chapter. The algorithm can reverse the processes of round key addition, byte substitution, row displacement, and column mixing executed during encryption. Therefore, the encryption algorithm in this chapter can be fully decrypted in reverse. Fig 4(b) illustrates the decryption process.

## 4 Experimental results and analysis

### 4.1 Simulation environment

To evaluate the encryption performance and security of the proposed algorithm in this chapter, as well as to verify its effectiveness on images with repetitive pixel content and demonstrate its applicability across diverse fields, we selected five representative images with distinct themes. These images span five domains: portrait (Female), animal (Baboon), medical (X-Ray), nature (Peppers), and grayscale (Gray). These images were utilized to simulate the proposed algorithm and compare it with existing encryption methods. The simulation experiments were conducted on a computer equipped with an Intel Core i7 2.6 GHz CPU and 16 GB RAM, running the Windows 10 operating system and MATLAB 2016b software environment. The comparative encryption algorithms were configured with parameters optimized to showcase their best performance, ensuring a fair and accurate evaluation.

### 4.2 Histogram analysis

**Fig 8:**
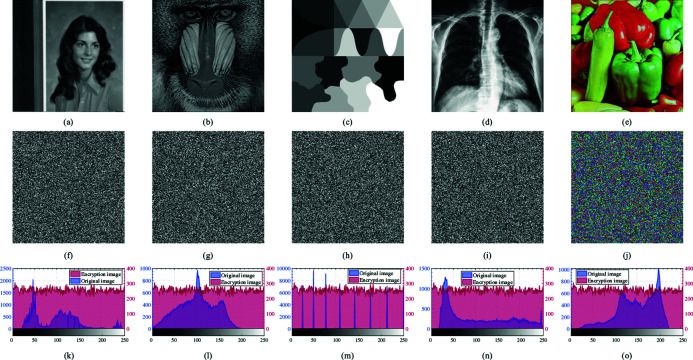
Histogram analysis results. (a–e) Original images of each test image. (f–j) Encrypted images of each test image. (k–o) Histograms before and after encryption of each test image.

A histogram provides a visual representation of pixel value distribution within an image. In images containing discernible information, pixel values typically cluster, resulting in an irregular and uneven histogram. Encrypted images, however, which are imbued with random noise and lack visible information, should exhibit uniform pixel value distribution without any recognizable pattern. Effective encryption algorithms aim to disrupt these clusters, transforming pixel values into randomly distributed classes that result in a uniform histogram. Analyzing histogram distributions of encrypted images serves as a key indicator for assessing encryption efficacy. Fig 8 illustrates histograms of five test images before and after encryption using the algorithm presented in this chapter.

The graphs reveal significant differences in pixel value distribution among pre-encryption images of similar content, characterized by distinct clustering. In contrast, histograms of encrypted images exhibit consistent distribution patterns across different image types. Encrypted image histograms show no discernible clustering, demonstrating stable pixel value distribution curves where each value occurs uniformly throughout the image. Thus, the encryption algorithm proposed in this chapter effectively mitigates pixel aggregation in images, thereby safeguarding the contained information.

### 4.3 Information entropy analysis

Entropy serves as a quantitative metric to characterize the degree of chaos within a system, where higher entropy values correspond to greater system chaos. In the context of information theory, entropy quantifies the uncertainty inherent in a dataset. Systems with volatile internal data exhibit higher entropy values, while stable systems exhibit lower entropy values. In unencrypted images intended for human perception, pixel value distributions typically display high local similarity among adjacent pixels to render recognizable visual information. Increased similarity in pixel values leads to lower entropy values. Thus, entropy serves as a quantitative measure to assess the presence of local similarity in images, thereby quantifying the degree to which information can be visually perceived. Effective encryption algorithms aim to reduce local similarity in images while ensuring accurate decryption of the original content, thereby enhancing chaos in encrypted images. In this study, information entropy is employed to evaluate encryption algorithm performance, reflecting the degree of chaos in images. The calculation method is illustrated as follows:

$$H=-\sum_{k}p_{k}\log_{2}(p_{k})$$ (11)

where *P*_*k*_ denotes the probability of internal elements within the information system having a value *k*. For 256 order grayscale images, the ideal probability of an any specific value *k* is *P*_*k*_=1/256. The theoretical maximum value is calculated as follows:

$$H=-\sum_{k}\frac{1}{256}\log_{2}(\frac{1}{256})=8$$ (12)

Table 2 presents the information entropy data for the test images before and after encryption using the algorithm described in this chapter, along with comparisons to other similar algorithms. Analysis of Table 2 reveals significant disparities between the information entropy of various images before encryption and the theoretical maximum value of 8. However, post-encryption using the proposed algorithm, the information entropy of the encrypted results approaches a level very close to 8. This indicates that the algorithm effectively enhances pixel confusion in images. Specifically, for Female images encrypted using different algorithms, the information entropy value after applying the algorithm in this chapter reaches 7.9980, demonstrating superior encryption efficacy compared to similar algorithms.

**Table 2:** Information entropy analysis results.

Image	Algorithm	Plaintext	Ciphertext
**Baboon**	TDHCS	7.3739	7.9976
**Texmos3**	TDHCS	3.7649	7.9977
**X-Ray**	TDHCS	7.5575	7.9985
**Peppers R Channel**	TDHCS	7.3031	7.9979
**Peppers G Channel**	TDHCS	7.5585	7.9985
**Peppers B Channel**	TDHCS	7.0972	7.9982
**Female**	TDHCS	7.1325	7.9982
	Ref [31]	7.1325	7.9975
	Ref [32]	7.1325	7.9973
	AES		7.9975

### 4.4 Pixel correlation analysis

**Fig 9:**
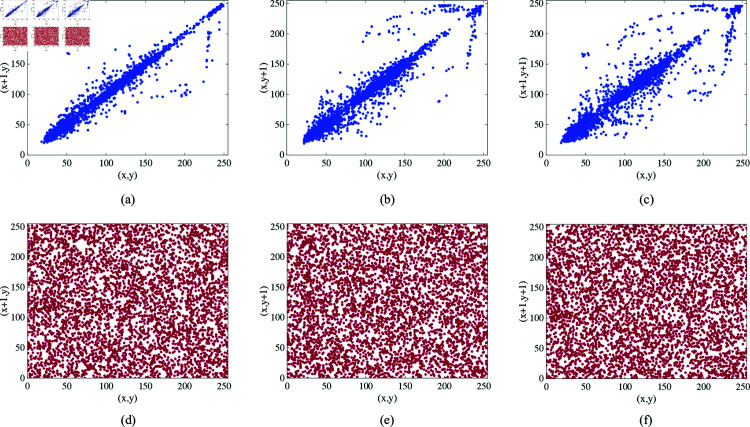
The distributions of adjacent pixels of Female image. (a–c) The distributions of the original image in the horizontal, vertical, and diagonal directions, respectively. (d–f) The distributions of the encrypted image in the horizontal, vertical, and diagonal directions, respectively.

Adjacent pixels in the original image exhibit correlations in various directions, and the objective of encryption algorithms is to minimize these correlations effectively. To assess the algorithm’s capability to reduce correlations between adjacent pixels in images, the following formula is employed for quantitative correlation calculation:

$$\rho (x,y)=-\frac{1}{N}\sum_{k}^{N}\frac{\left(x_{i}-E(x))(y_{i}-E(y)\right)}{\sqrt{D(x)}\sqrt{D(y)}}$$ (13)

where *N* represents the logarithm of the number of selected pixel pairs for calculation, *x* and *y* denote two sets of pixels selected for calculation, *E*(*x*) denotes the mean of the pixel array, and *D*(*x*) denotes the variance of the pixel array. The calculation range of ρ(x,y) is [0,1]. A value closer to 1 indicates a stronger correlation between pixels, whereas a value closer to 0 indicates weaker correlation. Taking the Female image as an example, 5,000 pairs of pixels were randomly selected from the horizontal, vertical, and diagonal directions of the image, and their pixel value distribution is shown in Fig 9.

Selecting various test images, we applied the algorithm described in this chapter for encryption. We randomly selected 5000 pairs of pixels from three directions (horizontal, vertical, and diagonal) of both the unencrypted and encrypted images, and calculated their correlations. The results are summarized in Table 3. Prior to encryption, the pixel correlations in different image types were consistently close to one. Post-encryption, correlations in all directions decreased to approximately zero. This study demonstrates the algorithm’s effective ability to reduce correlations between adjacent pixels in images.

**Table 3:** Correlation coefficient values between adjacent pixels before and after encryption.

Image	Horizontal Plain	Horizontal Cipher	Vertical Plain	Vertical Cipher	Diagonal Plain	Diagonal Cipher
**Female**	0.9872	0.9700	0.9553	0.0022	–0.0053	0.0050
**Baboon**	0.7457	–0.0006	0.7932	0.0018	0.6890	–0.0007
**Texmos3**	0.9853	–0.0018	0.9909	0.0026	0.9807	0.0066
**X-Ray**	0.9912	–0.0051	0.9863	0.0018	0.9796	0.0060
**Peppers R Channel**	0.9649	0.0020	0.9593	–0.0045	0.9261	0.0029
**Peppers G Channel**	0.9748	–0.0004	0.9680	0.0083	0.9420	–0.0033
**Peppers B Channel**	0.9506	–0.0035	0.9515	–0.0024	0.9219	0.0019

Table 4 presents a comparison of the results between the algorithm proposed in this chapter, the traditional AES algorithm, and other similar algorithms, focusing on the correlation between adjacent pixels for the Female image. From the table, it is evident that the algorithm in this chapter achieves the lowest correlation among encrypted image pixels. This indicates that compared to the algorithms examined, the approach detailed in this chapter effectively minimizes the correlation between adjacent pixels, thereby achieving superior encryption effectiveness.

**Table 4:** Comparison of correlation coefficient values with existing schemes for Female image.

Algorithm	Ref [22]	Ref [33]	Ref [34]	AES	TDHCS
**Horizontal**	–0.0158	0.0062	0.0026	0.0241	0.0025
**Vertical**	–0.0118	0.0033	0.0050	0.0241	0.0005
**Diagonal**	0.0004	–0.0023	0.0020	0.0017	0.0047

### 4.5 Differential-attack analyses

Differential attack is a method of attacking the key of an encryption algorithm by constructing plaintext or ciphertext with specific differences. If the correlation between the encryption operation and the plaintext image is not sufficiently low during the encryption process, it becomes challenging to defend against differential attacks. Superior encryption algorithms should effectively withstand various forms of differential attacks, ensuring that even for specially constructed plaintext images, the resulting encryption exhibits no obvious, clustered, or recognizable patterns.

**Fig 10:**
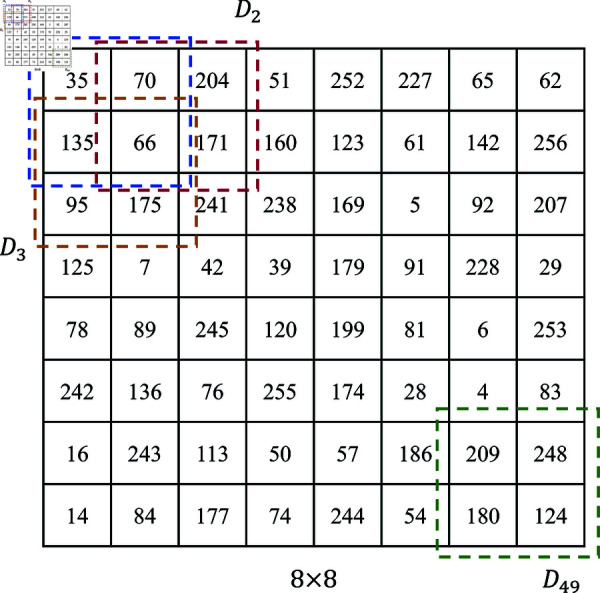
Example of image segmentation during BACI calculation process.

To quantitatively evaluate encryption algorithms’ resistance to differential attacks, three key metrics are commonly employed: Number of Pixels Change Rate (NPCR), Uniform Average Change Intensity (UACI), and Block Average Change Intensity (BACI). The analysis involves modifying specific pixels in the encrypted image, separately encrypting the modified and unmodified versions, and computing these indicators for comparison. NPCR, UACI, and BACI range from [0, 100], with theoretical values of 99.6094%, 33.4635%, and 26.7712%, respectively. When the calculated results for two images are close to these theoretical values, they can be considered statistically similar. Let *C*_1_ and *C*_2_ denote two encrypted images, each of size M×N. The calculation methods for these three indicators are as follows:

1. NPCR:
  $$NPCR(C_{1},C_{2})=\sum_{i=1}^{M}\sum_{j=1}^{N}\frac{\left|Sign[C_{1}(i,j),C_{2}(i,j)]\right|}{M\times N}\times 100\%$$ (14)
  The calculation method of the *Sign*(*x*,*y*) function sign is as follows:
  $$\text{Sign}(x,y)=\left\{ \begin{array}{cc} 0 & x=0\\ 1 & x\neq 0 \end{array}\right.$$ (15)
2. UACI:
  $$UACI(C_{1},C_{2})=\sum_{i=1}^{M}\sum_{j=1}^{N}\frac{\left|C_{1}(i,j)-C_{2}(i,j)\right|}{M\times N\times 255}\times 100\%$$ (16)
3. BACI:
  Firstly, divide the image into K=(M−1)(N−1) small squares each of size 2×2. An example of this segmentation is illustrated in Fig 10.

The *k*_*th*_ small block is:
  $$\text{Sign}(x,y)=[\begin{array}{cc} d_{k1} & d_{k2}\\ d_{k3} & d_{k4} \end{array}]$$ (17)
where *m*_*k*_ represents the average difference between any two elements in a small square, calculated as follows:
  $$m_{k}=\frac{\left|d_{k1}-d_{k2}\right|+\left|d_{k1}-d_{k3}\right|+\left|d_{k1}-d_{k4}\right|+\left|d_{k2}-d_{k4}\right|+\left|d_{k3}-d_{k4}\right|}{6}$$ (18)
  Then BACI can be calculated using the method of Eq 19:
  $$\text{BACI}(P_{1},P_{2})=\frac{\sum_{k=1}^{\left(M-1)(N-1\right)}m_{k}}{(M-1)(N-1)\times 255}$$ (19)

To evaluate the algorithm’s resistance to differential attacks in this chapter, random pixels are selected from the chosen test images, and their values are altered by adding or subtracting one. Subsequently, new images are generated from these modifications, and both the original and modified images are encrypted using the algorithm presented here. The NPCR, UACI, and BACI values between the encrypted images are then computed. This process is repeated 500 times, and the average values for each indicator are summarized in Table 5.

**Table 5:** The NPCR, UACI, and BACI values of various images.

Image	Type	Min (%)	Max (%)	Mean (%)
**Female**	NPCR	99.4095(–0.1999)	99.7086(+0.0991)	99.6081(–0.0013)
	UACI	33.1637(–0.2997)	33.6473(+0.1837)	33.4458(–0.0176)
	BACI	26.5784(–0.1927)	26.9494(+0.1781)	26.7507(–0.0204)
**Baboon**	NPCR	99.5453(–0.0641)	99.6735(+0.0641)	99.6085(–0.0009)
	UACI	33.2649(–0.1986)	33.7254(+0.2619)	33.5068(+0.0433)
	BACI	26.5823(–0.1889)	26.9687(+0.1975)	26.8112(+0.0400)
**Texmos3**	NPCR	99.5438(–0.0656)	99.6826(+0.0732)	99.6107(+0.0013)
	UACI	33.1857(–0.2778)	33.6059(+0.1424)	33.4179(–0.0456)
	BACI	26.5301(–0.2411)	26.9075(+0.1363)	26.7429(–0.0283)
**X-Ray**	NPCR	99.5529(–0.0565)	99.6674(+0.0580)	99.6074(–0.0020)
	UACI	33.1643(–0.2992)	33.6813(+0.2178)	33.4419(–0.0216)
	BACI	26.4976(–0.2736)	26.8967(+0.1255)	26.7463(–0.0249)
**Peppers R Channel**	NPCR	99.5483(–0.0611)	99.6796(+0.0702)	99.6084(–0.0010)
	UACI	33.1678(–0.2957)	33.6952(+0.2317)	33.4352(–0.0283)
	BACI	26.5600(–0.2112)	26.9431(+0.1719)	26.7513(–0.0199)
**Peppers G Channel**	NPCR	99.5483(–0.0611)	99.6719(+0.0625)	99.6098(+0.0004)
	UACI	33.1920(–0.2715)	33.6709(+0.2074)	33.4419(–0.0216)
	BACI	26.6109(–0.1603)	26.9107(+0.1395)	26.7564(–0.0148)
**Peppers B Channel**	NPCR	99.5438(–0.0656)	99.6841(+0.0747)	99.6092(–0.0002)
	UACI	33.1843(–0.2792)	33.6806(+0.2171)	33.4327(–0.0308)
	BACI	26.5637(–0.2075)	26.9823(+0.2111)	26.7514(–0.0198)

It is evident that after encryption using the algorithm in this chapter, images with minimal differences exhibit NPCR, UACI, and BACI values very close to their theoretical counterparts, suggesting randomness akin to two random images.

The results of differential analysis between this chapter’s algorithm and traditional AES algorithm and other similar algorithms are presented in Table 6, using Female as the test image. From the table, it is evident that for the same image, the algorithm in this chapter achieves NPCR, UACI, and BACI values closest to their theoretical counterparts. This indicates that the algorithm in this chapter is highly sensitive to minor changes in the plaintext and effectively enhances information security against differential attacks.

**Table 6:** Comparison of NPCR, UACI, BACI values of various algorithms for Female image.

Algorithm	NPCR (%)	UACI (%)	BACI (%)
**Ref [35]**	99.6197(+0.0103)	33.5282(+0.0647)	25.6879(-1.0833)
**Ref [36]**	99.5999(–0.0095)	33.5060(+0.0425)	26.3701(–0.4011)
**Ref [37]**	99.6026(+0.0068)	30.3008(+3.1629)	26.9855(+0.2143)
**AES**	99.6136(+0.0042)	33.4545(–0.0089)	26.7568(–0.0144)
**TDHCS**	** 99.6063(−0.0031) **	** 33.4681(−0.0046) **	** 26.8000(+0.0288) **

### 4.6 Algorithm efficiency analysis

Encryption algorithms not only need to effectively protect the data of images, but also need to improve the encryption efficiency of encryption algorithms as much as possible. For the test image of size, the complexity of the algorithm in the chaotic sequence generation stage is, and the complexity in the encryption stage is. As only one round of encryption operation is required, the time complexity of the encryption algorithm in this chapter is. In order to further evaluate and compare the encryption efficiency of the algorithms in this chapter, different sizes of test images were selected, and different algorithms were used for encryption testing in the same simulation environment. The encryption time, encryption throughput (ET), and number of cycles per byte (NCPB) required for each bit were calculated and compared. The calculation methods for ET and NCPB are as follows:

$$\left\{ \begin{array}{c} ET=\frac{\text{image file size}(MB)}{\text{encryption time}(seconds)}\\ NCPB=\frac{CPU_{speed}(Hertz)}{ET(Bps)} \end{array}\right.$$ (20)

For Female images of different sizes, different algorithms were used to encrypt 100 times, and the average results are shown in Table 7.

**Table 7:** Comparison result of the encryption efficiency required by different encryption algorithms for the Female image at varying sizes.

Algorithm	Image size	Encryption time (s)	Decryption time (s)	ET (MBps)	NCPB
**Ref [21]**	256×256	4.3360	4.5786	0.0144	201,190,400
	512×512	17.5132	18.0340	0.0142	203,153,120
	1024×1024	63.3452	63.5972	0.0157	183,701,080
**Ref [38]**	256×256	2.4432	3.3491	0.0255	113,364,480
	512×512	9.5006	11.4679	0.0263	110,206,960
	1024×1024	41.6909	41.8852	0.0239	120,903,610
**Ref [39]**	256×256	10.4200	10.8923	0.0059	483,488,000
	512×512	43.9594	44.7659	0.0056	509,929,040
	1024×1024	192.6695	195.3441	0.0051	558,741,550
**AES**	256×256	17.3090	18.7009	0.0036	803,137,600
	512×512	69.1949	69.1034	0.0036	802,660,840
	1024×1024	269.8310	273.4527	0.0037	782,509,900
**TDHCS**	256×256	** 2.2369 **	** 2.5931 **	** 0.0279 **	** 103,792,160 **
	512×512	** 8.9222 **	** 9.1036 **	** 0.0280 **	** 103,497,520 **
	1024×1024	** 32.7017 **	** 32.9117 **	** 0.0305 **	** 94,834,930 **

From the table, it is evident that across various image sizes and metrics, the encryption efficiency of the algorithm presented in this chapter shows substantial improvements compared to traditional AES, and it also demonstrates strong performance relative to similar algorithms.

## 5 Conclusions

To ensure algorithmic security, traditional AES encryption methods typically require a total of 13 encryption rounds for image encryption. While this iterative process enhances encryption security, it also increases the computational resource consumption for both encryption and decryption. Leveraging the compatibility of hyperchaotic systems with image encryption due to their sensitivity to initial values and inherent randomness, this study enhances and simplifies the traditional AES algorithm using the TDHCS. The original key expansion process is replaced by chaotic sequences generated through hyperchaotic mapping, which exhibit strong randomness. This improvement not only enhances encryption randomness but also simplifies the encryption steps and reduces processing time. The proposed dynamic scrambling and diffusion method achieves secure encryption of plaintext images with only a single encryption round.

Experimental simulations and statistical analyses demonstrate that the proposed algorithm offers a large key space and high sensitivity, effectively resisting common cryptographic attacks such as statistical analysis, differential analysis, and plaintext analysis. The correlation coefficient of adjacent pixels in the encrypted images approaches the theoretical value of 0. Furthermore, the encrypted images exhibit NPCR, UACI, and BACI values close to their theoretical ideals of 99.6094%, 33.4635%, and 26.7712%, respectively. Additionally, the significant improvement in encryption efficiency validates the algorithm’s applicability to digital image encryption processes.

Experimental simulations and statistical analyses demonstrate that the proposed algorithm offers a large key space and high sensitivity, effectively resisting common cryptographic attacks such as statistical analysis, differential analysis, and plaintext analysis. The correlation coefficient of adjacent pixels in the encrypted images approaches the theoretical value of 0. Furthermore, the encrypted images exhibit NPCR, UACI, and BACI values close to their theoretical ideals with only minimal deviations of 0.0031%, 0.0046%, and 0.0288%. Additionally, the significant improvement in encryption efficiency validates the algorithm’s applicability to digital image encryption processes. Specifically, the proposed algorithm achieves an 87.08% improvement in encryption efficiency compared to traditional AES, and a 48.41% improvement compared to other AES-based chaotic encryption algorithms.

However, despite the promising results obtained through experimental simulations for both the chaotic system and the encryption algorithm, certain limitations remain. The improved AES algorithm still requires further optimization of the encryption process to reduce encryption time and maximize encryption efficiency. Moreover, the robustness of the proposed encryption scheme against advanced cryptanalysis techniques, such as machine-learning-based attacks, needs further investigation. Future research will focus on enhancing the chaotic sequence generation mechanism to improve key sensitivity and unpredictability. Additionally, optimizing the encryption framework for color images and real-time applications will be explored to expand its practical usability. Further studies will also investigate the potential of hybrid encryption models that integrate hyperchaotic encryption with quantum cryptographic techniques to achieve enhanced security and computational efficiency.
